# Effects of Betaine and Polydextrose on Intestinal Microbiota and Liver Ergothioneine in a High-Fat Diet-Fed Mouse Model and a Human Colonic Simulation Model

**DOI:** 10.3390/nu17010109

**Published:** 2024-12-30

**Authors:** Markku T. Saarinen, Sofia D. Forssten, Kara Evans, Kaisa Airaksinen, Rasmus Telving, Bettina Høj Hornshøj, Henrik Max Jensen, Jenna Jokkala, Kati Hanhineva, Kirsti Tiihonen

**Affiliations:** 1 IFF, Health & Biosciences, Sokeritehtaantie 20, 02460 Kantvik, Finland; 2 IFF, Health & Biosciences, 3329 Agriculture Drive, Madison, WI 53716, USA; 3 IFF, Health & Biosciences, Edwin Rahrs Vej 38, 8220 Brabrand, Denmark; 4 Institute of Public Health and Clinical Nutrition, School of Medicine, University of Eastern Finland, Yliopistonranta 1 B, 70211 Kuopio, Finland; 5 Food Sciences Unit, Department of Life Technologies, Finland and University of Turku, 20014 Turku, Finland

**Keywords:** dietary supplements, microbial metabolites, gut microbiome, diet-induced obesity, fiber, in vitro colonic fermentation

## Abstract

Background/Objectives: Ergothioneine (EGT) is an effective antioxidant that animals cannot produce and has an important anti-inflammatory role in cell protection, which can help lower the risk of various diseases. In this study, we investigated the potential role of gut microbiota in the production of EGT, which was found to increase in the mouse liver after dietary supplementation with betaine (BET) or polydextrose (PDX). Methods: The effects of BET and PDX on the gut microbiota and tissue EGT content were investigated using a diet-induced obese mouse model and simulated fermentation in the human colon. Male C57BL/6J mice were fed a high-fat diet (HFD) for 8 weeks to induce obesity and related metabolic disorders, and for the last 4 weeks of this study, the mice continued on the same diet, supplemented with BET, PDX, or their combination. The potential function of BET and PDX in microbial EGT production was further studied in an in vitro human colon model. Results: The quantity of *Bifidobacterium* spp. and Bacteroidota were significantly higher in the feces of mice on diets supplemented with PDX or BET + PDX, and Enterobacteriaceae levels were significantly higher in PDX-supplemented mice than in HFD-fed mice. Untargeted metabolomic analysis of the liver revealed a significant increase in EGT in mice fed HFDs with BET or BET + PDX. Microbial analysis from samples collected from the human in vitro model showed significant changes in *Neglecta timonensis*, *Blautia faecis*, *Lachnospiracea incertae sedis*, *Faecalibacillus*, and *Stenotrophomonas maltophilia* species, along with an increase in microbial metabolites, namely, acetic, propionic and butyric acids, and a decrease in 2-methylbutyric acid. Conclusions: Although PDX and BET or their combination affected microbial composition and metabolites in the human colon simulation model, the model used was not able to detect a significant change in microbiota-based EGT production and, therefore, could not explain the increase in EGT in the liver of betaine-fed mice.

## 1. Introduction

Polydextrose (PDX) is a highly branched, randomly crosslinked glucose polymer that is used widely as a dietary fiber in the food industry. As a nondigestible food ingredient, PDX has demonstrated prebiotic potential based on its ability to alter intestinal microbial composition [1] and be partially metabolized by colonic microbes [2,3,4]. In clinical trials, PDX has had satiating effects [5,6], reducing postprandial serum triglyceride levels [7] and total body fat mass when combined with probiotics [8].

Betaine (BET, N,N,N-trimethylglycine) is an N-methylated quaternary amino acid derivative that is present especially in plants. As BET is obtained from various food sources, it is readily absorbed into circulation and distributed throughout specific tissues. In the body, BET is synthesized from choline and is involved in one-carbon metabolism as a methyl donor in many pathways and acts as an osmolyte in cells. In humans, a higher intake of betaine (BET) correlates with a more favorable body composition, but the exact mechanism of this relationship is unknown, although several paths have been suggested, including its effects on lipid metabolism and protein synthesis [9]. The administration of betaine to diet-induced obese mice was shown to improve glucose homeostasis, reduce hepatic lipid accumulation and dyslipidemia, alleviate inflammation, slow the development of obesity, and enhance whole-body energy expenditure [10]. BET supplementation also normalized high-fat diet (HFD)-induced dysbiosis of the gut microbiota and increased antiobesity species, such as *Akkermansia muciniphila*, *Lactobacillus sensu lato*, and *Bifidobacterium*, in these mice [10]. Moreover, in mice that lacked gut microbiota, BET prevented HFD-induced obesity and metabolic syndrome (MetS) and limited the inactivation of brown adipose tissue.

Ergothioneine (EGT) is a naturally occurring BET amino acid, a potential vitamin analog [11], and an effective antioxidant [12]. Only certain fungi, yeast, and bacteria can synthesize EGT, whereas animals and higher plants acquire EGT by transporter-mediated uptake [13]. Nevertheless, EGT is found in a wide range of foods, and most of the EGT in humans and other animals is believed to be attributed to the intake of plant-based material. Although EGT is present in small amounts in the diet, it has been detected in most human and animal tissues, including the liver, kidney, and red blood cells, indicating efficient absorption and retention of this nutrient. At high levels, EGT is considered an excellent cytoprotective agent against various types of cell damage, such as those caused by reactive oxygen or nitrogen species or UV radiation. It has also been suggested to be a beneficial antioxidant in inflammatory diseases, including acute respiratory distress syndrome and cardiovascular disease. [12]. Furthermore, among the numerous metabolites examined, higher blood EGT was found to be most significantly associated with a lower risk of cardiometabolic disease and mortality, independent of age, sex, nutritional status, alcohol consumption, or smoking status [13].

In this study, the effects of BET and the combination of BET and PDX on the gut microbiota were assessed by a diet-induced obese mouse model and the EnteroMix^®^ human colon in vitro model. Previous studies with the same mice model [14,15] have reported findings of an untargeted metabolite profiling analysis that examined metabolic changes in the liver, muscles, and adipose tissue and the expression of inflammatory markers that were associated with obesity in mice. The current study presents new metabolomic data on EGT levels in the liver of mice that were fed an HFD that was supplemented with BET or a combination of BET and PDX. The observed changes in hepatic EGT levels and microbiota, along with the proposed health benefits of EGT, prompted us to investigate further. We used an in vitro human colonic fermentation model with the same supplements to explore whether diet-induced microbial changes in the gut could influence the production of EGT and other metabolites by microbiota.

## 2. Materials and Methods

### 2.1. Animals and Diets

The experimental conditions and sampling procedures for the mouse study have been previously described [15]. The study protocol was reviewed and approved by the Animal Experiment Board of the Regional State Administrative Agency for Southern Finland in Hämeenlinna, Finland (approval number: ESAVI-2010-08343/Ym-23). In brief, 4-week-old male C57BL/6J mice were acclimatized for 1 week and fed a standard rodent chow (Teklad 2016S and 2018S, Envigo, Indianapolis, IN, USA) ad libitum, after which they were randomly divided into 5 groups (*n* = 10 per group, no significant difference between body weights) and fed a low-fat diet (LFD, D12450B with reduced cellulose content (0.6% *w*/*w*), Research Diets, Inc., New Brunswick, NJ, USA) for 4 weeks or a high-fat diet (HFD, D12451 with reduced cellulose content (0.6% *w*/*w*), Research Diets, Inc.) for 4 weeks to induce obesity and related metabolic disorders. The macronutrient energy content of the LFD and HFD is presented in Table 1. After 4 weeks, the LFD group continued with the same diet (no supplementation) for another 4 weeks. The HFD group was randomized into four groups (*n* = 7–10; no significant difference between body weights) to receive 1 of the following supplements for another 4 weeks: (1) HFD (no supplementation), (2) HFD + PDX (6.66% *w*/*v* Litesse^®^ Ultra™, Danisco USA Inc., Terre Haute, IN, USA), (3) HFD + BET (1% *w*/*v* Betafin^®^ BF 20, Finnfeeds Finland Ltd., Naantali, Finland), or (4) HFD + PDX (6.66% *w*/*v*) + BET (1% *w*/*v*). Each animal received the supplements daily, freely available in their drinking water. After two days, the PDX dose was reduced to 3.33% *w*/*v* due to reported loose stools in PDX-supplemented HFD groups. The daily dose of PDX and BET in mice was calculated to be proportional to the high dose that could be used in humans. This calculation is indicative and considers the size and metabolic activity of the animals and the levels used in previous animal studies in mice. The condition and welfare of the animals were monitored at minimum every 24 h when the feed and water supplies were checked and their consumption measured.

At the end of this study, the animals were CO_2_-stunned and decapitated after a 7 h fast. Muscle and liver samples were collected, rinsed with 0.9% NaCl, weighed, snap-frozen in liquid nitrogen, and stored at −80 °C. The small intestine was dissected, and the cecum and ileum were separated. For microbial analysis, digesta, mucus, and intestinal walls from both the cecum and ileum were collected as follows: First, the digesta was removed and placed into sterile pre-weighed test tubes on ice. Then, the tissue was cut open, rinsed with 0.9% NaCl, and attached to a rubber surface. The mucus layer was gently scraped with metal spatulas, and the mucus samples were placed on ice in pre-weighed ceramic bead tubes. The remaining ileal and caecal tissues were weighed and packed in aluminum foil for snap freezing in liquid nitrogen.

### 2.2. In Vitro Fermentation

The effects of BET, PDX, and BET + PDX on the human intestinal microbiota were analyzed in the EnteroMix^®^ semicontinuous colon simulator system as previously described [16]. To model the human gut microbiota, fresh stool samples were obtained from healthy adult volunteers to prepare the inoculum for the simulation. One simulator unit consists of four vessels (V1–V4) that model different compartments of the human colon from the proximal to the distal part, each with a different pH and flow rate. For the simulations, 0.2% and 1% BET, 2% PDX, and a combination of 1% BET and 2% PDX were prepared in simulation medium. The simulations were run for 48 h (*n* = 3), after which the fecal simulation slurries were collected from the vessels V1–V4. The samples were stored at −80 °C before analysis. The simulations with human fecal material were approved by the HUS Regional Committee on Medical Research Ethics at the Helsinki University Hospital (Approval number: HUS/1975/2021).

### 2.3. Microbiological Analysis of Mouse Digestive Tract

Microbial DNA was extracted from feces, intestinal digesta, and mucosal and intestinal tissues. The samples were weighed in tubes that contained VK01 glass or zirconia beads (Bertin Instruments, Montigny-le-Bretonneux, France). Next, 1 mL PBS (137 mM NaCl, 10 mM phosphate, 2.7 mM KCl; pH 7.4) was added, and the samples were disrupted twice with a Precellys bead beater (Bertin Instruments) for 3 × 30 s cycles at 6800 rpm. The resulting DNA was purified, extracted on an automated MagMAX™ Sample Preparation System (Life Technologies, Halle, Belgium) using the MagMAX™ Nucleic Acid Isolation Kit (AM1840, Life Technologies), and measured on a Nanodrop ND-1000 full-spectrum UV/VIS spectrophotometer (Thermo Fisher, Wilmington, DE, USA).

Quantitative polymerase chain reaction (qPCR) was performed to quantify *Bacteroidota* [17], Enterobacteriaceae [18], and Streptomyces spp. [19] by SYBR green method (Power SYBR Green kit, Applied Biosystems, Foster City, CA, USA), whereas *Bifidobacterium* spp. [20] and *Escherichia coli* [21] were analyzed by TaqMan (Applied Biosystems)-based chemistry. All qPCR assays were run in a total volume of 25 µL, containing 1 ng of template DNA. The DNA was amplified and detected on an ABI 7500 (Applied Biosystems). Standard curves were obtained from a 10-fold dilution series, ranging from 10 pg to 10 ng of DNA from standard cultures of *Bacteroides fragilis* ATCC 25285, *E. coli* DSM 30083, *Bifidobacterium lactis* Bi-07, and *Streptomyces albus* DSM 40313. DNA was measured in triplicate samples, and the mean quantity was calculated as log10 genomes/g wet weight.

### 2.4. Microbial Analysis of Colon Simulator Samples

Fecal microbial DNA was used to assess microbiota populations by 16S amplicon sequencing as described [22].

### 2.5. Nontargeted Metabolomic Profiling of Liver and Muscle Samples

Nontargeted metabolite profiling analysis of liver and muscle tissue samples was performed as previously described [15]. Briefly, 90% methanol (*v*/*v* H_2_O, LC-MS Ultra CHROMASOLV R, Fluka, Buchs, Switzerland) was added to 100 mg (±2 mg) of cryo-ground frozen liver or muscle tissue. The samples were shaken for 20 min and centrifuged for 10 min at + 4 °C (16,000× *g*). Supernatants were filtered using 0.2-µm syringe filters with a PTFE membrane (Acrodisc R, PALL Co., Port Washington, NY, USA) and stored at −20 °C until liquid chromatography–mass spectrometry (LCMS) analyses. EGT was identified by hydrophilic interaction chromatography (HILIC) combined with positive-mode electrospray (ES) ionization. Precursor ion *m*/*z* 230.096 gave MS/MS fragment ions 186.105, 127.032, 100.022, 60.081 with good fragment match with the EGT pure compound (L-(+)-ergothioneine, Sigma-Aldrich, St Louis, MI, USA) listed in the Metlin compound library.

### 2.6. Determination of EGT in Colon Simulator Samples by LCMS

The simulation samples were prepared as described [23] by adding 100 µL fecal slurry to a 1.5 mL microcentrifuge tube, followed by 100 µL acetonitrile (Sigma-Aldrich) and 50 µL of internal standard (ergothioneine-d9, Toronto Research Chemicals, Toronto, ON, Canada), 500 ng/mL in acetonitrile). The resulting solution was mixed briefly and centrifuged for 5 min at 14,000 rpm. Next, 150 µL of the supernatant was transferred to a vial, to which 50 µL 5-iodoacetamidofluorescein (5-IAF, Sigma-Aldrich) in 150 mM Na2HPO4 (pH 13, 400 µg/mL) was added and mixed. The solution was allowed to react for 30 min at room temperature (light-protected), after which 200 µL purified water (Milli-Q^®^, Merck, Darmstadt, Germany) was added, and the solution was mixed again before analysis by LCMS. Following the same procedure as above, a matrix-matched calibration curve was prepared by adding 100 µL acetonitrile, containing 10–500 ng/mL EGT, to a blank fecal slurry sample (100 µL). The LOQ was 1 ng/mL, and the recovery rate was 84% to 105%.

The LCMS analysis was performed on an Agilent Technologies 1290 ultra-high-performance liquid chromatograph (UHPLC, Waldbronn, Germany) that was coupled to a Thermo Fisher Scientific TSQ Vantage triple quadrupole MS with heated electrospray interface (HESI-II) (San Jose, CA, USA). A Waters Acquity UPLC HSS T3 column (1.8 µm, 100 × 2.1 mm id, Milford, MA, USA) was used for the separation. Chromatographic separation was performed at a column temperature of 35 °C using a mobile phase that consisted of (A) water/formic acid (Sigma-Aldrich) (1000/1 *v*/*v*) and (B) acetonitrile/formic acid (1000/1 *v*/*v*). The gradient was (%B/min) 25/0, 62.5/5, 90/5.1, 90/8, 25/8.1, and 25/11 at a flow rate of 0.3 mL/min. Samples were cooled to 10 °C in the autosampler, and the injection volume was 5 µL. The MS was operated in positive ionization mode with the following settings: capillary temperature: 300 °C; vaporizer temperature: 300 °C; sheath gas pressure: 45 units; ion sweep gas pressure: 0; auxiliary spray pressure: 5; spray voltage: 3500 V; probe position: C; and collision pressure: 1.5 mTorr. The SRM transitions and MS settings were as follows: for 5-IAF derivatized EGT- parent ion 309.1 *m*/*z*, product ions 287.1 *m*/*z* (quant ion, collision energy 15), 309.1 *m*/*z* (collision energy 5), and s-lens 75 and for 5-IAF derivatized ergothioneine-d9- parent ion 313.4 *m*/*z*, product ions 291.4 *m*/*z* (quant ion, collision energy 15), 313.4 *m*/*z* (collision energy 5), and s-lens 75.

### 2.7. Analysis of Short-Chain and Branched-Chain Fatty Acids

Short-chain fatty acids (SCFAs) and branched-chain fatty acids (BCFAs) in the fecal simulation slurries were analyzed by gas chromatography as previously described [24].

### 2.8. Statistical Analysis

Statistical significance between groups was calculated by one-way or two-way analysis of variance (ANOVA), followed by Bonferroni’s multiple comparison test with a 95% confidence level in GraphPad Prism, version 9, unless otherwise stated. Bacterial quantities in mouse intestinal digesta, feces, and mucosal and intestinal tissue samples were log-transformed. A *p*-value < 0.05 was considered significant.

Statistical analysis of intestinal microbiota was performed as described [22]. The R package ‘rmcorr’ (R Core Team, 2017) [25] was used to assess correlations between microbiota and metabolite production by repeated measures. Each simulator vessel was treated as a repeated measurement from an “individual.” *p*-values were adjusted for multiple comparisons by Benjamini–Hochberg FDR, as noted.

## 3. Results

### 3.1. Metabolomic Analysis of Liver and Muscle Samples

**Figure 1:**
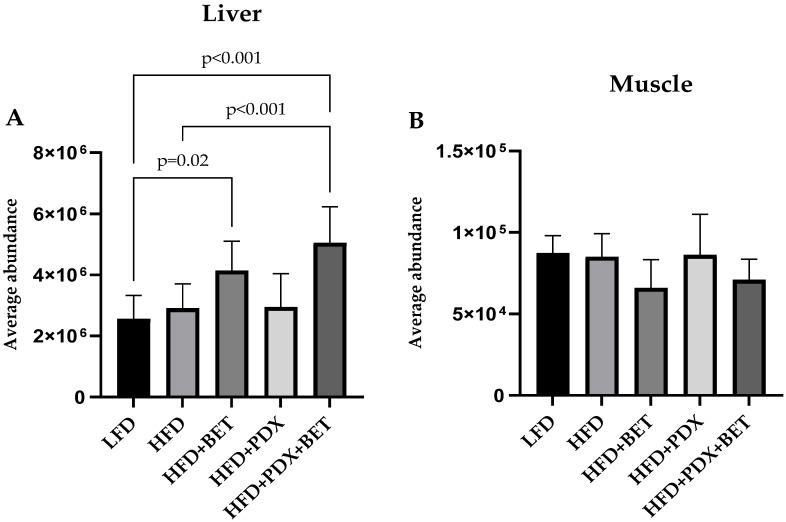
Relative abundance [mean + SD of relative peak area (*n* = 7–10 mice per group)] in ergothioneine (EGT) after a 4-week feeding period in (**A**) liver and (**B**) muscle, as measured by nontargeted liquid chromatography–mass spectrometry metabolite profiling. The mice were fed a low-fat diet (LFD) or a high-fat diet (HFD) supplemented with betaine (BET) (1% *w*/*v*), polydextrose (PDX) (3.33% *w*/*v*), or BET (1%) + PDX (3.33%) freely available in drinking water. Statistically significant differences between groups (*p* < 0.05) compared with the HFD and LFD are denoted.

As reported earlier [15], by nontargeted UHPLC-quadrupole time of flight mass spectrometry metabolite profiling, we observed an increase in several metabolites in the carnitine biosynthesis pathway in mice after supplementation with BET and BET + PDX in liver and muscle. Further, a statistically significant increase in EGT content was noted in the liver when the mice were fed a BET-containing HFD compared with an LFD (1.6-fold), as was seen with an HFD with BET + PDX versus an LFD or HFD (2.0-fold and 1.7-fold, respectively) (Figure 1). In muscle samples, no significant changes in EGT were detected between diets.

### 3.2. Microbiological Analysis of Mouse Gastrointestinal Tract

Bacterial quantities in mouse intestinal digesta, feces, and mucosal and intestinal tissue samples are listed in Table S1. A statistically significant decrease in *Bifidobacterium* spp. levels was observed in the feces of mice that consumed an HFD (*p* < 0.001) and HFD + BET (*p* < 0.01) compared with the LFD group. Compared with mice that were fed an HFD, *Bifidobacterium* spp. counts were significantly higher in mice that received HFD + PDX (*p* < 0.05) and HFD + BET + PDX (*p* < 0.001). Also, *Bifidobacterium* spp. levels increased in the group that was given an HFD that was supplemented with BET + PDX versus mice that received HFD + BET (*p* < 0.05). In cecal digesta, Bifidobacterium spp. decreased significantly in mice that were fed an HFD, HFD + BET, or HFD + PDX compared with the LFD (*p* < 0.05 for all). No significant differences in *Bifidobacterium* spp. counts were observed between the three HFDs. In the intestinal tissue of the cecum, *Bifidobacterium* spp. levels were significantly higher in the HFD + PDX + BET versus HFD + PDX group (*p* < 0.01).

Fecal *Enterobacteriaceae* content was significantly higher in mice that received HFD + BET (*p* < 0.01) and HFD + PDX (*p* < 0.001) compared with the LFD, as with the HFD + PDX group versus the HFD (*p* < 0.001). Moreover, *Enterobacteriaceae* declined significantly in the HFD + PDX + BET group compared with HFD + PDX (*p* < 0.001). In cecal digesta, *Enterobacteriaceae* decreased significantly only in the HFD + PDX + BET group versus mice that were fed HFD + PDX (*p* < 0.05). *E. coli* was not detected in any intestinal sample.

Bacteroidota rose significantly in the feces of mice that consumed HFD + PDX or HFD + PDX + BET versus the LFD (*p* < 0.001 for both), as it did in the HFD + BET, HFD + PDX, and HFD + BET + PDX groups compared with the HFD (*p* < 0.001 for all). Bacteroidota counts also increased significantly in the HFD + PDX and HFD + BET + PDX groups versus HFD + BET (*p* < 0.001 for both). In the cecum, Bacteroidota counts were significantly higher in the HFD + PDX and HFD + BET + PDX groups compared with the LFD (*p* < 0.01 for both) and HFD (*p* < 0.001 for both). Relative to the HFD + BET group, Bacteroidota counts also rose significantly in the HFD + PDX and HFD + BET + PDX groups (*p* < 0.05 and *p* < 0.01, respectively). In mucus that was obtained from the cecum, we noted a significant increase (*p* < 0.05) in Bacteroidota in the HFD + PDX group versus the HFD and HFD + BET, whereas in the intestinal tissue of the cecum, the only significant change that occurred was an elevation in *Bacteroidota* in the HFD + PDX + BET compared with HFD + PDX group (*p* < 0.05). In the intestinal tissue of the ileum, Bacteroidota increased significantly in the HFD + PDX versus LFD, HFD, and HFD + BET groups (*p* < 0.05 for all).

*Streptomyces* spp. was only detected in feces, decreasing significantly in animals that consumed HFD + BET compared with an LFD (*p* < 0.001), HFD (*p* < 0.01), HFD + PDX (*p* < 0.05), and HFD + BET + PDX (*p* < 0.001).

### 3.3. Microbiological Analysis of In Vitro Colon Simulation Samples

#### 3.3.1. α- and β-Diversity of Simulated Microbiota

**Figure 2:**
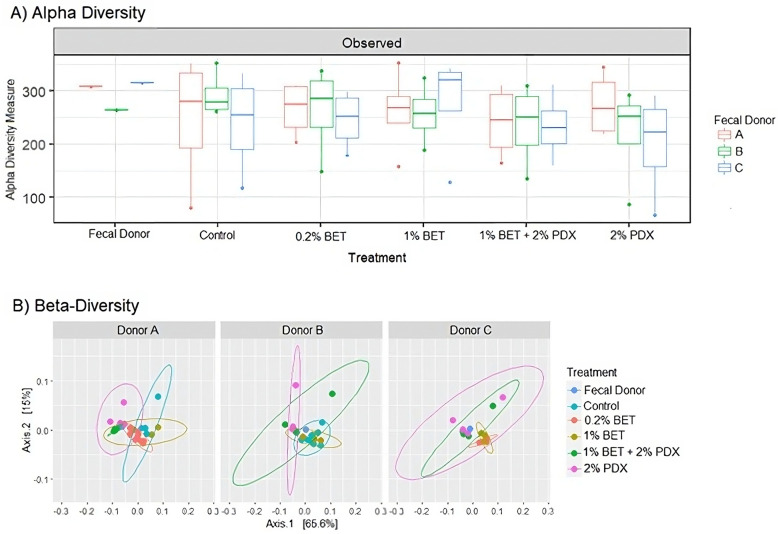
(**A**) α- and (**B**) β-diversities of each sample, grouped by fecal donor (A, B, or C). α-diversity was calculated based on the observed number of features. The center line in the boxplot indicates the mean of each group, and the box includes all samples in the first and third quartiles. The principal coordinate analysis (PCoA) plot of β-diversity was calculated by weighted UniFrac distance. Betaine, BET; polydextrose, PDX.

There was no difference in α-diversity between donors. However, donor (R^2^ = 0.238, *p* = 0.001) and group (R^2^ = 0.162, *p* = 0.001) had a significant effect on β-diversity between samples. The α- and β-diversities (weighted Unifrac distance) of the donors and groups are shown in Figure 2 (A and B, respectively).

#### 3.3.2. Microbiota Composition of In Vitro Colon Simulation Samples

**Figure 3:**
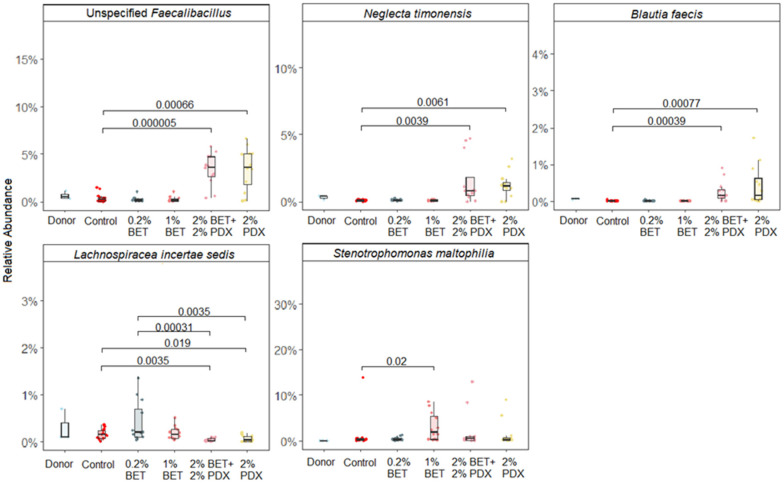
The relative abundance of species differed significantly compared with control after in vitro colon simulations. Betaine, BET; polydextrose, PDX.

Significant changes in the five species were observed among the microbial communities between groups compared with the simulation medium control. Faecalibacillus (4% relative abundance), Neglecta timonensis (1% relative abundance), and Blautia faecis (0.25% relative abundance) were significantly enriched in the 1% BET-2% PDX (*p* < 0.0001, *p* = 0.0046, and *p* < 0.0004, respectively) and 2% PDX (*p* < 0.001, *p* = 0.005, and *p* < 0.0008, respectively) groups versus the control. In contrast, Lachnospiracea incertae sedis (0.25% relative abundance) was less abundant in the 1% BET-2% PDX (*p* = 0.0035) and 2% PDX (*p* = 0.019) groups compared with the control. Stenotrophomonas maltophilia (2.5% relative abundance) was significantly enriched (*p* = 0.02) in the 1% BET group but not the 0.2% BET group versus the control. The changes in the relative abundance of these species between groups and the control are shown in Figure 3.

### 3.4. Microbial Metabolites in Colon Simulation Samples

**Figure 4:**
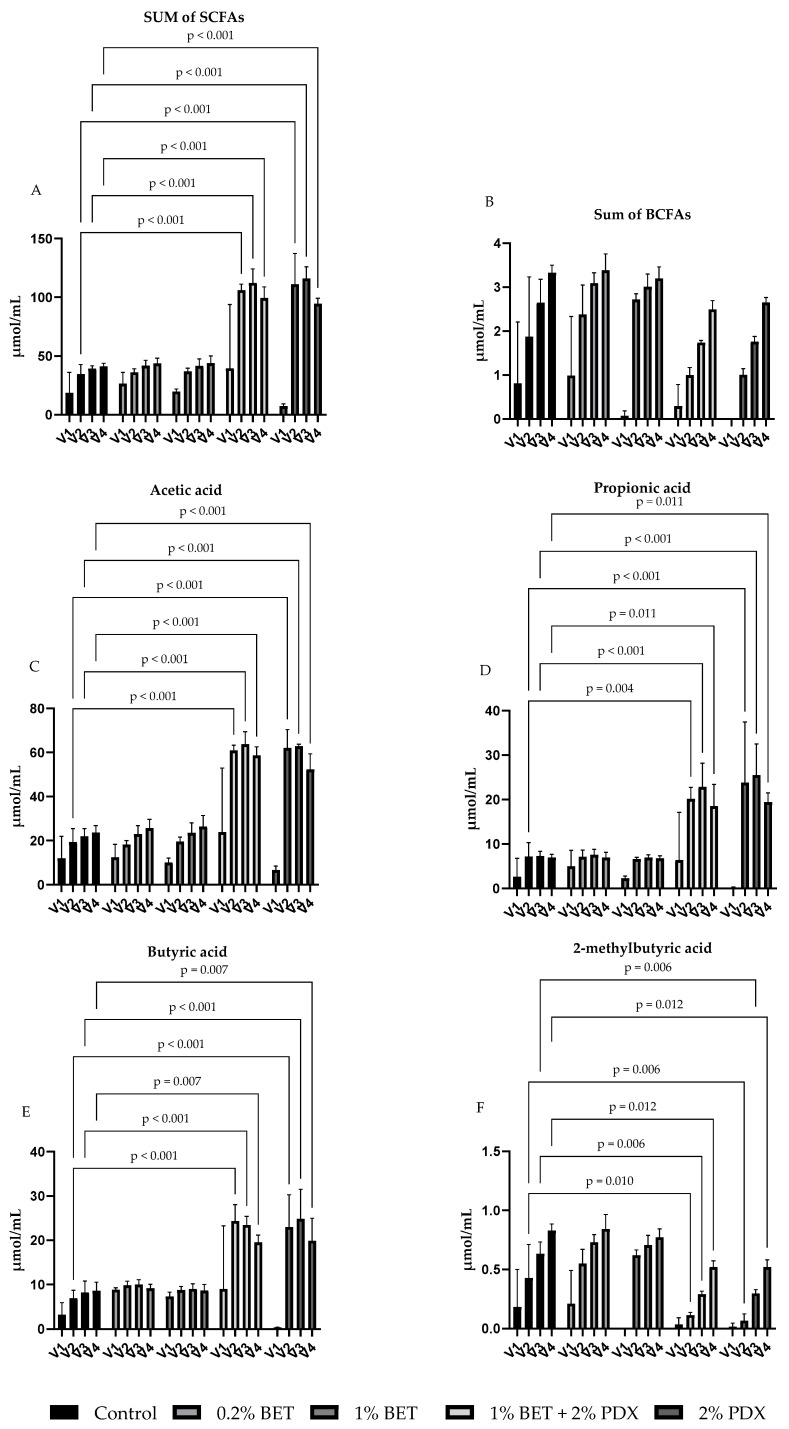
Levels of short-chain fatty acids (SCFAs) and branched-chain fatty acids (BCFAs) [as mean (µmol/mL) + SD of 3 fermentations] in fecal slurry samples in simulator vessels V1–V4 (representing different parts of the colon) in the following groups: betaine (BET) (0.2% and 1%), polydextrose (PDX) (2%), and a combination of BET (1%) and PDX (2%) added to the simulation medium (control). Concentrations of the sum of SCFAs (**A**); sum of BCFAs (**B**); and acetic (**C**), propionic (**D**), butyric (**E**), and 2-methylbutyric acid (**F**).

After the in vitro colonic fermentation, SCFAs (acetic acid, butyric acid, propionic acid, and valeric acid) and BCFAs (2-methylbutyric acid, isobutyric acid, and isovaleric acid) were measured to examine the effects of 0.2% and 1% BET, 2% PDX, and their combination (1% BET + 2% PDX) on bacterial metabolism versus control. In general, total SCFA concentrations in vessels V1–V4 ranged from 4.8 to 132 µmol/mL, depending on the substrate, and total BCFA concentrations ranged from below the limit of detection (0.2 µmol/mL) to 3.6 µmol/mL (Figure 4).

Compared with the control, the fermentations with 2% PDX and the 1% BET + 2% PDX combination significantly increased the sum of SCFAs and the concentrations of acetic acid, propionic acid, and butyric acid in V2–V4 (Figure 4). Especially, the concentrations of acetic acid, propionic acid, and butyric acid rose equally with these substrates, and the relative proportions of these metabolites, expressed as percentages, did not change versus the control. In the fermentations with 2% PDX and 1% BET + 2% PDX, the production of 2-methylbutyric acid declined in V2–V4 (Figure 4). The fermentations with 0.2% BET and 1% BET as substrate did not lead to significant changes in any metabolite.

### 3.5. Correlation Between Microbiota and Metabolites in the Colon Simulator Samples

To better understand the relationship between the microbiota in the simulated colon and the metabolites they produce, correlation analysis between genus-level microorganisms and acidic end-products was performed. The most abundant taxa (*Segatella copri*, *Collinsella aerofaciens*, *Gemmiger formicilis*, and *Phascolarctobacterium faecium*) were not significantly associated with any SCFA or BCFA. However, five strains had a strong or weak correlation with these metabolites (Table 2). *B. faecis*, *Faecalibacillus*, and *N. timonensis* had robust, positive correlations with acetic, propionic, and butyric acid. *N. timonensis* also had a weak, positive correlation with isobutyric and isovaleric acid. *A. muciniphila* had strong, positive correlations with isovaleric acid, isobutyric acid, and 2-methylbutyric acid and a weak, positive association with acetic acid and valeric acid. *S. maltophila* correlated strongly but negatively with all acids.

### 3.6. EGT in Colon Simulation Samples

**Figure 5:**
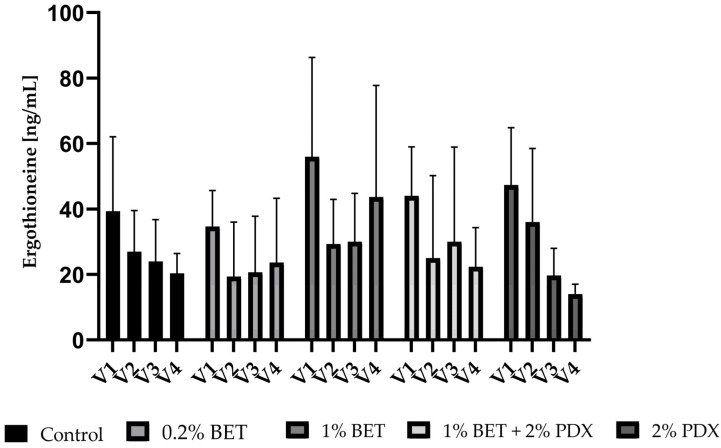
Production of ergothioneine after in vitro colonic fermentation in vessels V1–V4. The substrates betaine (BET) (0.2% and 1%), polydextrose (PDX) (2%), and a combination of BET (1%) + PDX (2%) were added to the simulation medium, which was also used as control. The concentrations are presented as mean (ng/mL) + SD of 3 fermentations.

After in vitro colonic fermentation, the concentration of EGT was analyzed in V1–V4. Quantifiable amounts of EGT were observed on all substrates and the control (Figure 5) and ranged from 5 to 91 ng/mL. No significant differences in EGT production were observed between groups.

## 4. Discussion

This study aimed to investigate the effects of BET and the combination of BET + PDX on the intestinal microbiota using an HFD-induced obese mouse model and to present a novel finding that follows up a published untargeted metabolite profiling analysis of mouse liver and muscle samples. Also, the slowed absorption of BET (e.g., through cereals that contain BET) in the colon was modeled by supplementing the EnteroMix^®^ colon simulator with BET and assessing the changes in microbial levels, microbial metabolites, and ERG content in various segments of the human colon.

EGT cannot pass through cell membranes without the EGT transporter ETT (OCTN1); thus, it has been used to estimate the levels and distribution of EGT in animals [26,27]. The expression of ETT varies between tissues and is high in the kidney, but elevated EGT levels are also found in red blood cells, the liver, and the spleen [13]. In our study, the changes in hepatic EGT levels constitute the first reported findings on the use of BET, alone or in combination with PDX, as a dietary supplement. Male mice were chosen for this study because variations in hormone levels, such as those due to the menstrual cycle, may cause variations in the results and complicate their interpretation. To generalize the increase in EGT levels in the liver due to the same supplements, the experiment should also be repeated with female mice. In muscle tissue, EGT did not differ significantly between any of the groups but was 29 and 34 times higher in the liver compared with muscle in the LFD and HFD groups, respectively. Its specific physiological function, localization, and minimum required amount in humans and other animals are unknown [12].

Biosynthesis of EGT has been observed in mycobacteria and fungi, but some *Actinomycetales*, *Actinomycetota*, and Pseudomonadota are able to produce EGT [28,29,30]. However, no study has fully demonstrated that intestinal bacteria synthesize EGT [26]. Among bacteria, *E. coli* has been shown to utilize EGT as a nitrogen source for growth [31]. Another study has reported that Helicobacter pylori acquire EGT from the host environment using EgtUV, a highly selective ATP-binding cassette transporter. In this comparative sequence analysis, EgtUV was generally conserved in gut microbes and widely distributed in Bacillota [32]. In a depressed rat model [33], *Limosilactobacillus reuteri* was suggested to produce EGT because changes in intestinal microbiota and sulfur metabolites correlated between increased fecal EGT and elevated *L. reuteri* levels, although no differences were observed in plasma EGT levels. A separate in vitro study examined whether *L. reuteri* can produce EGT [34], finding that upregulation of EGT in the bacteria or the correlation of increased EGT levels with *L. reuteri* in fecal samples is not direct evidence of EGT biosynthesis because the presence of EGT in a microorganism is not conclusive proof of EGT production, given that certain bacteria can obtain EGT from their environment. In silico analyses have indicated that Bacteroidota, Actinomycetota, and Pseudomonadota possess egtBD genes or homologs [26], and cloning egtBD genes into *E. coli* confers the ability to produce EGT to these bacteria [35].

In our study, Bacteroidota levels rose significantly in all samples in mice that were fed with HFD supplemented with BET or PDX, as expected, given that the genus Bacteroidota includes the most common degraders of dietary fiber. Previous studies in humans and mice have linked obesity to a decrease in bacteroidota [36,37]. Fiber increases fecal bifidobacteria content, and we noted a significant rise in bifidobacteria in mice that consumed HFD + PDX and HFD + BET + PDX compared with those on an HFD, with bifidobacterial levels closer to that with the LFD. In a separate study in which PDX was administered to mice on a Western diet (WD), Parabacteroides and Bifidobacterium were more abundant in WD + PDX-fed mice versus those on a WD alone [38]. PDX also promoted the growth of beneficial microbes, such as *Bacteroidota*, *Parabacteroides*, *Alloprevotella*, *Muribaculum*, *Akkermansia*, and *Ruminococcaceae*, in a study on obese mice [39]. In our report, no correlation between changes in EGT in the liver and gut microbiota was found. BET supplementation [10] improves HFD-induced dysbiosis of the gut microbiota and increases antiobesity strains, such as Bifidobacteria. *Enterobacteriaceae*, which were also predominated in another study in HFD-fed mice [40], increased significantly with HFD + PDX in our study but not with HFD + PDX + BET.

Because the samples in our mouse experiments were unavailable for sequencing, the effects of BET and PDX were evaluated using the EnteroMix^®^ human colon simulator. In fecal slurry samples from the simulation, the most highly represented taxa did not correlate significantly with any SCFA. *P. faecium* is an abundant colonizer of the human gastrointestinal tract, and *Phascolarctobacterium* can produce SCFAs and is associated with the metabolic state of the host [41]. *S. copri* is more prevalent in non-Western populations, likely due to its association with high-fiber LFDs. However, because the *S. copri* lineage consists of at least four distinct species-level lines, the conflicting observations regarding *S. copri* and human health are likely attributed to strain-specific metabolic diversity and dietary preferences [42]. *Collinsella* species are known for their ability to ferment a wide range of carbohydrates [43].

Significant differences in abundance were observed for *B. faecis*, *N. timonensis*, *Lachnospiracea incertae sedis*, *Faecalibacillus*, and *S. maltophilia*, depending on the group. *Blautia* has specific functions in biotransformation and crosstalk with other intestinal microorganisms [44,45]. In our study, supplementation with PDX increased the levels of *B. faecis*, correlating well with certain SCFAs. In contrast, PDX decreased the abundance of *L. incertae sedis*, although *Lachnospiraceae* produces SCFAs from dietary fiber [46].

It is possible that some microorganisms can degrade or use EGT to their advantage. As EGT is commonly found in the mammalian diet, the EgtUV transporter may have been acquired through lateral gene transfer to aid microbial adaptation to the host environment. The EgtUV transporter is also widely distributed among gastrointestinal microbes, and dietary EGT regulates the microbial redox in the gut, suggesting that interspecies competition for dietary EGT may have implications for human health [32]. Other biomes, such as the virome [47] and mycobiome [48,49], correlate significantly with health outcomes, meriting their analysis in future studies.

## 5. Conclusions

Supplemental betaine and polydextrose alter gut microbiota, as was demonstrated in both obese mice and a simulated human colon model. However, the human colonic simulation model was unable to detect a significant change in microbiota-based EGT production and thus could not explain the increase in EGT observed in the liver of betaine-fed mice. In order to verify the proposed health benefits and mechanisms of action of EGT, a natural antioxidant found in various organisms, studies using isotopically labeled precursors with more accurate analytical methods may be needed to confirm EGT production in gut biomes. This confirmation could also provide deeper insights into EGT’s role in human health and its potential therapeutic applications.

## Figures and Tables

**Table 1:** Macronutrient content in the low-fat diet (LFD) and high-fat diet (HFD) expressed as energy of total energy (%). The diets were enriched with Mineral Mix S10026 and Vitamin Mix V10001 (Research Diets, Inc.). In the HF diet, protein consisted mainly of casein, carbohydrates of sucrose (50%), maltodextrin (30%), corn starch (20%), fat of lard (88%), and soybean oil (12%).

	LFD	HFD
Protein	20	20
Carbohydrate	70	35
Fat	10	45
Fiber % (*w*/*w*)	0.5	0.6
Total	100	100
kcal/g	4.02	4.99

**Table 2:** Correlations between strains/species and short-chain fatty acids and branched-chain fatty acids. The correlation was considered to be strong when R-value > 0.5 and *p*-value < 0.001 (green cells) and weak when R-value < 0.5 and *p*-value < 0.05 and >0.001 (grey cells).

		*Neglecta timonensis*	*Blautia faecis*	*Faecali-bacillus*	*Stenotrophomo-nas maltophilia*	*Akkermansia muciniphila*
Acetic acid	*p*-value	<0.001	<0.001	<0.001	<0.001	0.03
	R-value	0.75	0.59	0.82	−0.73	0.32
Propionic acid	*p*-value	<0.001	<0.001	<0.001	<0.001	0.1
	R-value	0.66	0.68	0.8	−0.74	0.24
Butyric acid	*p*-value	<0.001	<0.001	<0.001	<0.001	0.17
	R-value	0.68	0.63	0.82	−0.7	0.2
Valeric acid	*p*-value	0.015	0.88	0.75	<0.001	0.002
	R-value	0.37	0.02	0.05	−0.49	0.47
Isobutyric acid	*p*-value	0.006	0.62	0.54	<0.001	<0.001
	R-value	0.42	−0.08	0.1	−0.58	0.66
Isovaleric acid	*p*-value	0.015	0.85	0.34	<0.001	<0.001
	R-value	0.37	−0.03	0.14	−0.60	0.64
2-methyl-butyric acid	*p*-value	0.055	0.97	0.58	<0.001	<0.001
	R-value	0.3	−0.007	0.09	−0.54	0.64

## Data Availability

The data that support the findings of this study are available from the corresponding author upon reasonable request and following approval of the institutional review board.

## Supplementary Materials

The following supporting information can be downloaded at https://www.mdpi.com/article/10.3390/nu17010109/s1. Table S1: Mean bacterial levels (expressed as log10 genomes/g and SD) in mouse intestinal digesta, feces, and mucosal and intestinal tissue samples by qPCR. The mice were fed an LDF or HFD or an HFD supplemented with BET, PDX, or a combination of BET + PDX in drinking water for 4 weeks. One-way ANOVA and Bonferroni’s multiple comparison test were used to calculate the differences between groups; values with different superscript letters differ significantly at *p* < 0.05.
