# Supplementary material for: Effects of Betaine and Polydextrose on Intestinal Microbiota and Liver Ergothioneine in a High-Fat Diet-Fed Mouse Model and a Human Colonic Simulation Model
Source: Nutrients. 2024 Dec 30;17(1):109. doi: 10.3390/nu17010109 (PMC11722985; doi:10.3390/nu17010109)
Supplement: Supplementary file 1 [file nutrients-17-00109-s001.zip › nutrients-3354533-supplementary.pdf]

Table S1.

Mean bacterial levels (expressed as log10 genomes/g and SD) in mouse intestinal digesta, feces, and mucosal and intestinal tissue samples by qPCR. The mice were fed an LDF or HFD or an HFD supplemented with BET, PDX, or a combination of BET+PDX in drinking water for 4 weeks. One-way ANOVA and Bonferroni's multiple comparison test were used to calculate the differences between groups; values with different superscript letters differ significantly at $p < 0.05$.

	LFD	HFD	HFD+BET	HFD+PDX	HFD+PDX+BET
	N	N	N	N	N
	Log$^{gen.}$/g (SD)	Log$^{gen.}$/g (SD)	Log$^{gen.}$/g (SD)	Log$^{gen.}$/g (SD)	Log$^{gen.}$/g (SD)
Enterobacteriaceae					
Ileal digesta	3	4	3	7	2
	3.91 (0.26)	3.79 (0.59)	3.54 (0.19)	3.85 (0.48)	3.34 (0.46)
Cecal digesta	9	10	9	10	10
	5.60 (0.36)	5.86 (0.24)	5.86 (0.21)	5.94 (0.29)^{a}	5.54 (0.26)^{b}
Feces	9	10	10	10	10
	5.29 (0.19)^{a}	5.46 (0.23)^{ab}	5.68 (0.31)^{bc}	5.93 (0.22)^{c}	5.39 (0.21)^{abd}
Ileal mucus	5	5	5	5	5
	0.16 (0.23)	0.23 (0.16)	0.31 (0.09)	0.14 (0.16)	0.14 (0.09)
Cecal mucus	5	5	5	5	3
	0.24 (0.10)	0.25 (0.17)	0.17 (0.15)	0.18 (0.13)	0.40 (0.10)
Ileal tissue	9	7	10	6	9
	6.79 (0.35)	6.92 (0.67)	6.97 (0.26)	6.98 (0.36)	6.54 (0.46)
Bifidobacteria					
Ileal digesta	6	1	1	8	1
	7.40 (0.35)	5.16 (0.00)	7.16 (0.00)	6.39 (0.25)	6.88 (0.00)
Cecal digesta	10	10	10	10	10
	9.22 (0.24)^{a}	8.53 (0.42)^{b}	8.60 (0.70)^{b}	8.60 (0.31)^{b}	8.73 (0.55)
Feces	10	10	10	10	10
	9.26 (0.35)^{a}	8.12 (0.40)^{b}	8.43 (0.80)^{bd}	8.79 (0.43)^{acd}	9.09 (0.43)^{ac}
Ileal mucus	5	4	3	4	4
	1.36 (0.99)	0.11 (0.16)	1.08 (0.89)	0.76 (0.67)	2.46 (3.78)
Cecal mucus	5	4	5	5	4
	2.20 (2.28)	0.14 (0.13)	0.16 (0.12)	0.38 (0.30)	0.57 (0.62)

Ileal tissue	9	3	7	6	7
	7.83 (0.47)	6.53 (1.37)	7.51 (0.49)	7.12 (0.71)	6.88 (1.18)
Cecal tissue	10	4	8	10	10
	6.25 (0.49)	5.97 (0.72)	6.22 (0.55)	5.43 (0.44)^{a}	6.50 (0.97)^{b}
Bacteroidota					
Ileal digesta	4	1	1	8	1
	5.99 (0.43)	5.34 (0)	4.93 (0)	6.23 (0.98)	6.85 (0)
Cecal digesta	10	10	10	10	10
	9.29 (0.25)^{a}	9.20 (0.20)^{a}	9.30 (0.11)^{a}	9.59 (0.16)^{b}	9.61 (0.21)^{b}
Feces	10	10	10	10	10
	8.75 (0.25)^{a}	8.50 (0.33)^{a}	8.90 (0.22)^{c}	9.89 (0.17)^{bd}	9.91 (0.17)^{bd}
Ileal mucus	5	5	5	5	5
	0.07 (0.09)	0.04 (0.04)	0.06 (0.03)	1.71 (2.83)	0.37 (0.29)
Cecal mucus	5	5	5	5	4
	1.21 (1.00)	0.34 (0.36)^{a}	0.38 (0.28)^{a}	6.65 (6.26)^{b}	3.26 (2.70)
Ileal tissue	9	7	10	6	9
	6.46 (0.38)^{a}	6.53 (0.59)^{a}	6.57 (0.38)^{a}	7.46 (0.70)^{b}	6.80 (0.79)
Cecal tissue	10	5	9	10	10
	6.93 (0.50)	6.82 (0.67)	6.80 (0.46)	6.62 (0.36)^{a}	7.42 (0.74)^{b}
Streptomyces					
Feces	10	10	10	10	10
	7.45 (0.14)^{a}	6.20 (2.22)^{a}	2.07 (3.34)^{b}	5.36 (2.89)^{a}	6.90 (0.46)^{a}

N, number of samples analyzed; Log$^{gen.}$, base 10 logarithm of number of genomes detected; SD, standard deviation; qPCR, quantitative polymerase chain reaction; LFD, low-fat diet; HFD, high-fat diet; BET, betaine; PDX, polydextrose.
